# Medical College Notes

**Published:** 1890-07

**Authors:** 


### Medcal College Notes.

THE Medical Department of Niagara University has begun the erection of a large addition to their building on Ellicott street. The plans for this new building are very complete and practical, and will largely increase the teaching advantages of this school.

---

THE Marion-Sims Medical College has been founded in St. Louis. Among the list of professors we note the names of Drs. I. N. Love and Y. H. Bond, which may afford our readers an idea of the excellence of this new teaching body. These names would not be found in improper company.

---

THE COLLEGE OF PHYSICIANS AND SURGEONS OF CHICAGO.—This finely appointed Medical School building is well equipped with laboratories, and affords ample opportunity for the pursuit of histological, physiological and chemical studies. Attention is invited to its announcement on advertising page xxvii.

THE College of Physicians and Surgeons of Baltimore is out with its announcement, which may be found on advertising page xxvii. This college furnishes many advantages to the student not obtained elsewhere, and its faculty, headed by Professor Thomas Opie, M. D., as Dean, is one of the strongest in the country.

---

HARVARD UNIVERSITY.—Summer courses in the Harvard Medical School, 1890.—The Medical School of Harvard University has arranged a series of courses to be given by the instructors in the Harvard Medical School during the summer of 1890. As some of the classes are necessarily limited in number, students will be received in the order of their application. Certificates of attendance will be given to students who may desire them. For further information address the secretary, Dr. W. F. Whitney, Harvard Medical School, Boylston street, Boston, Mass.

